# Diterpenoids with Anti-Inflammatory Activity from the Wood of *Cunninghamia konishii*

**DOI:** 10.3390/molecules18010682

**Published:** 2013-01-04

**Authors:** Yu-Chang Chen, Yen-Cheng Li, Bang-Jau You, Wen-Te Chang, Louis Kuoping Chao, Lee-Chiang Lo, Sheng-Yang Wang, Guan-Jhong Huang, Yueh-Hsiung Kuo

**Affiliations:** 1 Department of Chinese Pharmaceutical Sciences and Chinese Medicine Resources, College of Pharmacy, China Medical University, Taichung 404, Taiwan; 2 Department of Chemistry, National Taiwan University, Taipei 106, Taiwan; 3 Department of Cosmeceutics, College of Pharmacy, China Medical University, Taichung 404, Taiwan; 4 Department of Forestry, National Chung Hsing University, Taichung 402, Taiwan; 5 Tsuzuki Institute for Traditional Medicine, China Medical University, Taichung 404, Taiwan

**Keywords:** *Cunninghamia konishii*, Taxodiaceae, diterpenoid, 7,20-dinorabietane, konishone, 3β-hydroxy-5,6-dehydrosugiol, anti-inflammatory

## Abstract

Two new diterpenoids, konishone (**1**) and 3β-hydroxy-5,6-dehydrosugiol (**2**), along with three known diterpenoids—hinokiol (**3**), sugiol (**4**), and 12-hydroxy-6,7-secoabieta-8,11,13-triene-6,7-dial (**5**)—were isolated from the wood of *Cunninghamia konishii*. Compound **1** is a novel skeleton of the 7,20-dinorabietane-type diterpene. In addition, when RAW264.7 macrophages were treated with different concentrations of compounds **1**, **3**, and **5** together with LPS, a significant concentration-dependent inhibition of NO production was detected. The IC_50_ values for inhibition of nitrite production of compounds **1**, **3**, and **5** were about 9.8 ± 0.7, 7.9 ± 0.9, and 9.3 ± 1.3 μg/mL, respectively. This study presents the potential utilization of compounds **1**, **3**, and **5**, as lead compounds for the development of anti-inflammatory drugs.

## 1. Introduction

**Figure 1:**
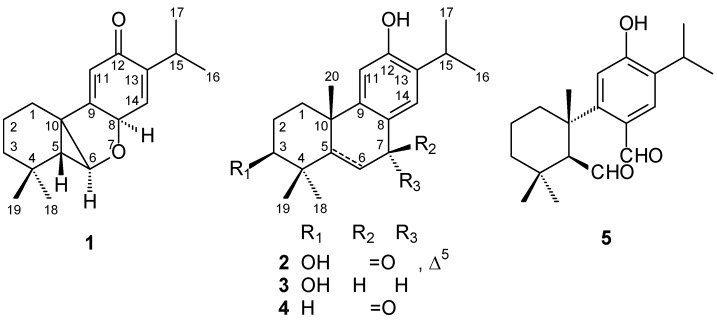
The chemical structures of compounds **1**–**5**.

*Cunninghamia konishii* Hayata (Taxodiaceae), one of the *Cunninghamia* which has two species in eastern Asia, is an endemic coniferous tree distributed in the northern and central part of Taiwan at altitudes of 1,300–2,700 m [1]. The wood of this tree is one of the best building materials available in Taiwan. The chemical components of wood [2,3,4,5,6,7,8,9], bark [10], leaf [8], and whole plant [11] of *C. konishii* have been reported. Some isolates of this plant exhibit antifungal activity [8,9] and cytotoxicity [11]. In connection with our interest in the components of this plant, a further chemical study on the same EtOAc-soluble fraction led to the isolation of two new diterpenoids, konishone (**1**) and 3β-hydroxy-5,6-dehydrosugiol (**2**), along with three known diterpenoids, namely hinokiol (**3**), sugiol (**4**), and 12-hydroxy-6,7-secoabieta-8,11,13-triene-6,7-dial (**5**) (Figure 1). The isolation and structural elucidation of these new compounds and the anti-inflammatory activity of the isolates are described herein.

## 2. Results and Discussion

**Figure 2:**
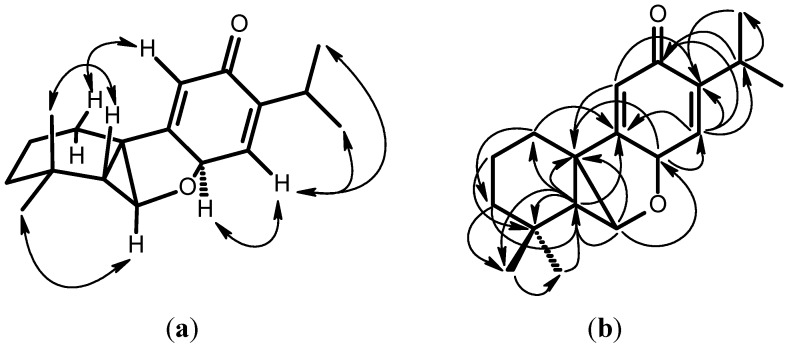
Key NOESY contacts (**a**) and HMBC connectivities (**b**) of compound **1**.

Konishone (**1**) was obtained as yellowish oil. Its molecular formula was established as C_18_H_24_O_2_ by the HRMS peak at *m*/*z* 272 [M^+^]. The presence of a cross conjugated dienone was revealed by the UV (237, 260 nm), IR spectrum data (3078, 1653, 1626, 1602 cm^−1^), ^1^H-NMR (Table 1) [δ 6.06 (s, 1H), 6.31 (s, 1H)], and ^13^C-NMR (Table 1) [δ 187.1 (C=O), 161.9, 149.6, 138.5, 126.5]. The ^1^H-NMR spectrum (Table 1) showed an isopropyl group [δ 1.05 (d, *J* = 6.0 Hz, H-16), 1.07 (d, *J* = 6.0 Hz, H-17), 2.96 (sept, *J* = 6.0 Hz, H-15)] attached on a double bond. Two methyl group signals at δ 1.00 (H-19) and 1.21 (H-18) showing HMBC correlations with δ 34.5 (C-4) suggested the presence of a geminal dimethyl group. Two oxygenated carbon signals at δ 79.7 (C-6) and 67.5 (C-8) have corresponding proton signals at δ 4.61 (d, *J* = 10.3 Hz, H-6) and 4.11 (s, H-8), respectively. H-8 showed a HMBC correlation with C-6, C-10, C-11 and C-14, and H-6 showed HMBC correlations with C-5, C-8 and C-10. The result suggested that C-6 was linked to C-8 by an ether linkage. H-5 expressed HMBC correlations with C-1, C-3, C-4, C-6, C-10, C-18, and C-19. The molecular formula C_18_H_24_O_2_ indicated index of hydrogen deficiency (IHD) of seven. According to the above evidence and the HMBC correlations, konishone was proposed to be a 7,20-dinorabietane diterpene. By using AM1 theoretical calculations, the most stable conformation has the angles of H-C_5_-C_6_-H and H-C_8_-C_14_-H being 136.5° and 87.6°, respectively. The result agreed to the coupling constant of H-5 and H-6 (*J* = 10.3 Hz); H-8 and H-14 (singlet). According to the above data, the structure of konishone was elucidated as **1**, which was further confirmed by COSY, NOESY (Figure 2), ^13^C-NMR (Table 1), DEPT, HMQC and HMBC (Figure 2) experiments. This is a novel 7,20-dinorabietane-type diterpene skeleton.

**Table 1:** ^1^H- (400 MHz) and ^13^C-NMR (100 MHz) data (CDCl_3_) of compounds **1** and **2**. Chemical shifts δ in ppm relative to TMS, *J* in Hz.

Position	1		2	
	δ_H_	δ_C_	δ_H_	δ_C_
1	1.42 (m, 1H), 1.72 (m, 1H)	38.1	1.63 (ddd, 13.6, 3.6 , 3.6, 1H)	34.5
			2.13 (ddd, 13.6, 3.6, 3.6, 1H)	
2	1.58 (m, 1H), 1.72 (m, 1H)	18.1	1.95 (m, 1H), 2.01 (m, 1H)	27.2
3	1.20 (m, 1H), 1.41 (m, 1H)	42.9	3.39 (dd, 11.6, 4.8, 1H)	76.5
4	–	34.5	–	43.2
5	1.33 (d,10.3, 1H)	52.5	–	171.0
6	4.61 (d, 10.3, 1H)	79.7	6.51 (s, 1H)	125.7
7	–	–	–	185.1
8	4.11 (s, 1H)	67.5	–	129.3
9	–	161.9	–	153.3
10	–	56.0	–	40.6
11	6.31 (s, 1H)	126.5	6.82 (s, 1H)	111.1
12	–	187.1	–	157.5
13	–	149.6	–	133.7
14	6.06 (s, 1H)	138.5	7.98 (s, 1H)	125.0
15	2.96 (sept, 6.0, 1H)	26.3	3.16 (sept, 6.8, 1H)	26.9
16	1.05 (d, 6.0, 3H)	21.6	1.25 (d, 6.8, 3H)	22.3
17	1.07 (d, 6.0, 3H)	22.4	1.28 (d, 6.8, 3H)	22.5
18	1.21 (s, 3H)	21.3	1.33 (s, 3H)	22.4
19	1.00 (s, 3H)	34.8	1.28 (s, 3H)	27.4
20	–	–	1.49 (s, 3H)	32.3

**Figure 3:**
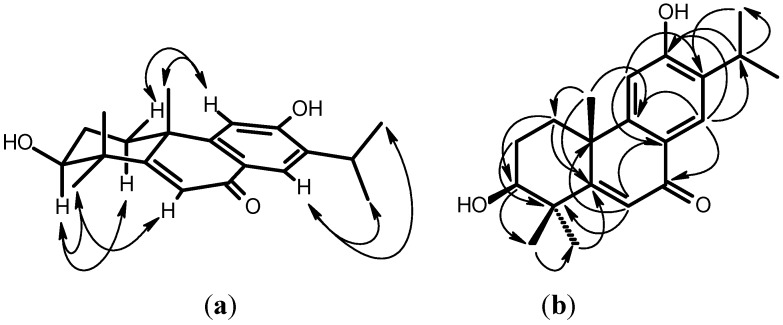
Key NOESY contacts (**a**) and HMBC connectivities (**b**) of compound **2**.

3β-Hydroxy-5,6-dehydrosugiol (**2**) was obtained as a yellowish amorphous solid. Its molecular formula was established as C_20_H_26_O_3_ by the HRMS peaks at *m*/*z* 314 [M^+^]. Absorptions in the IR spectrum were attributable to a hydroxy (3363 cm^−1^), a conjugated carbonyl (1639 cm^−1^), and a benzene ring (1613, 1502 cm^−1^). The ^1^H- and ^13^C-NMR spectra (Table 1) showed three singlet methyl groups [δ 1.33 (H-18), 1.28 (H-19), 1.49 (H-20)], an isopropyl group [δ 1.25 (d, *J* = 6.8 Hz, H-16), 1.28 (d, *J* = 6.8 Hz, H-17), 3.16 (sept, *J* = 6.8 Hz, H-15)] attached on a phenyl group [δ 6.82 (s, H-11), 7.98 (s, H-14), 129.3 (C-8), 153.3 (C-9), 111.1 (C-11), 157.5 (C-12), 133.7 (C-13), 125.0 (C-14)], and a conjugated enone [δ 6.51 (s, H-6), 185.1 (C-7), 171.0 (C-5), 125.7 (C-6)]. The ^1^H-NMR spectrum (Table 1) of **2** was similar to that of 5,6-dehydrosugiol [12], except for a hydroxy group at C-3. The proton resonating at δ 3.39 (dd, *J* = 11.6, 4.8 Hz) was attributable to H-3 with an α-axial orientation and was geminal to the hydroxy group. According to the above data, the structure of 3β-hydroxy-5,6-dehydrosugiol was elucidated as **2**, which was further confirmed by COSY, NOESY (Figure 3), ^13^C-NMR (Table 1), DEPT, HMQC and HMBC (Figure 3) experiments.

The three known compounds, hinokiol (**3**) [13], sugiol (**4**) [14], and 12-hydroxy-6,7-secoabieta-8,11,13-triene-6,7-dial (**5**) [15], were readily identified by comparison of physical and spectroscopic data (UV, IR, ^1^H-NMR, [α]_D_, and mass spectrometry data) with values found in the literature.

RAW264.7 is a mouse macrophage cell line used to model macrophage-mediated inflammatory events *in vitro*; thus, RAW264.7 cells were used to assess the effect of the isolated compounds on NO synthesis. The effect of compounds **1**, **3**, **4**, and **5** on RAW264.7 cell viability was determined by a MTT assay. Cells cultured with compounds **1**, **3**, and **4** at the concentrations (0, 2.5, 5, and 10 μg/mL) or compound **5** (0, 2.5, 5, 10, and 20 μg/mL) used in the presence of 100 ng/mL LPS for 24 h did not change cell viability (Table 2).

Compounds **1**, **3**, **4**, and **5** did not interfere with the reaction between nitrite and Griess reagents at 10 or 20 μg/mL (data not shown). Unstimulated macrophages, after 24 h of incubation in culture medium produced background levels of nitrite. When RAW264.7 macrophages were treated with different concentrations of compounds **1**, **3**, and **5** together with LPS (100 ng/mL) for 24 h, a significant concentration-dependent inhibition of nitrite production was detected. The IC_50_ values for inhibition of nitrite production of compounds **1**, **3**, and **5** were about 9.8 ± 0.7, 7.9 ± 0.9, and 9.3 ± 1.3 μg/mL. There was either a significant decrease in the nitrite production of group treated with **1**, **3**, and **5** (10 μg/mL) when compared with the LPS-alone group (*p* < 0.01).

**Table 2:** Cell viability and effect of compounds **1**, **3−5** on LPS-induced NO production in macrophages ^a^.

Compound	Dose (μg/mL)	Cell viability (% of control)	NO level	NO inhibition (% of control)	IC_50 _(μg/mL)
Control	(−)	93.0 ± 4.9	−0.5 ± 0.1	(−)	
LPS	(+)	98.7 ± 8.0	45.4 ± 2.7 ^###^	(−)	
1	2.5	103.7 ± 2.0	39.0 ± 1.2	14.2 ± 2.7	9.8 ± 0.7
	5	101.1 ± 3.1	34.6 ± 1.1 *	23.9 ± 2.5	
	10	98.9 ± 6.7	22.2 ± 1.1 **	51.0 ± 2.4	
	20	34.6 ± 9.6	(−)	(−)	
3	2.5	96.2 ± 1.5	39.3 ± 3.7	13.5 ± 8.2	7.9 ± 0.9
	5	87.1 ± 1.5	32.5 ± 0.6 *	28.5 ± 1.4	
	10	82.6 ± 1.5	16.0 ± 0.7 **	64.8 ± 1.5	
	20	75.8 ± 2.5	(−)	(−)	
4	2.5	90.4 ± 1.7	44.9 ± 1.9	1.2 ± 4.2	>20
	5	83.1 ± 1.8	40.5 ± 1.3	10.8 ± 2.9	
	10	81.8 ± 1.0	28.7 ± 2.1 *	36.9 ± 4.7	
	20	53.8 ± 1.9	(–)	(–)	
5	2.5	98.0 ± 1.2	38.0 ± 1.1	16.2 ± 2.4	9.3 ± 1.3
	5	100.4 ± 4.5	33.0 ± 1.3	27.4 ± 2.8	
	10	91.1 ± 2.0	21.1 ± 2.3 **	53.5 ± 5.1	
	20	82.2 ± 1.2	11.1 ± 4.4***	75.6 ± 9.7	

^a^ The data were presented as mean ± S.D. for three different experiments performed in triplicate;^ ###^ compared with sample of control group; * *p* < 0.05, ** *p* < 0.01, and *** *p* < 0.001 were compared with LPS-alone group.

## 3. Experimental

### 3.1. General

Silica gel *60 F_254_* precoated plates (*Merck*). Column chromatography (CC): Silica gel (*Merk* 70–230 mesh). High performance liquid chromatography (HPLC): *LDC Analytical-III* system; column: *LiChrosorb Si 60*, 7 μm, 250 × 10 mm. M.p.: *Yanaco-MP-S3* micro-melting-point apparatus; uncorrected. Optical rotation: *Jasco-DIP-180* polarimeter; in CHCl_3_. UV Spectra: *Hitachi-S-3200* spectrophotometer; *λ*_max_ (log *ε*) in nm; in MeOH. IR Spectra: *Perkin-Elmer-983G* FT-IR spectrophotometer; *ν* in cm^−1^. ^1^H-, ^13^C-, and 2D-NMR Spectra: *Varian-Unity-Plus-400* spectrometers; *δ* in ppm rel. to SiMe_4_, *J* in Hz. EI-MS and HR-EI-MS: *Jeol-JMS-HX300* mass spectrometer; *m*/*z* (rel. %).

### 3.2. Plant Material

The wood of *C. konishii* was collected at Luantashan, Nantau County, Taiwan, in December 1996 and was identified by Prof. Shao-Shun Ying (Department of Forestry, National Taiwan University). A voucher specimen (013492) has been deposited at the Herbarium of the Department of Botany, National Taiwan University, Taipei, Taiwan.

### 3.3. Extraction and Isolation

Dried wood (6.5 kg) of *C. konishii* was crushed into pieces and extracted with MeOH (60 L) three times (7 days each time) at room temperature. After removal of the solvent under vacuum, the extract (60.2 g) was suspended in water (500 mL) and successively partitioned into *n*-hexane (500 mL × 3), EtOAc (500 mL × 4), and *n*-BuOH (500 mL × 3). The EtOAc fraction (15.6 g) was chromatographed by silica gel column chromatography (using hexane–EtOAc and EtOAc–MeOH mixtures as solvent systems). Elution with *n*-hexane–EtOAc (7:3), (2:3), and (3:7) gave crude **4**, **3**, and **2**, respectively, while crude compounds **1** and **5** were eluted with *n*-hexane–EtOAc (9:1). Furture purification by HPLC gave **1** (2.5 mg), **2** (2.5 mg), **3** (14.0 mg), **4** (7.3 mg), and **5** (5.0 mg) using *n*-hexane–CH_2_Cl_2_–EtOAc (10:5:1), *n*-hexane–EtOAc–*i*-PrOH (15:5:1), CH_2_Cl_2_–acetone–*i*-PrOH (30:5:1), *n*-hexane–CH_2_Cl_2_–EtOAc–*i*-PrOH (50:10:5:1), and *n*-hexane–CH_2_Cl_2_–EtOAc (10:5:1), respectively.

### 3.4. Konishone (**1**)

Yellowish oil; [α]^27^_D_ +27.9° (*c* 0.23, CHCl_3_); UV (MeOH) *λ*_max_ (log *ε*): 237 (4.06), 260 (4.13) nm; IR (neat) *ν*_max_: 3078 (C–H, vinyl), 1653 (C=O, conjugated), 1626, 1602 (C=C), 1374, 1268 cm^−1^; EIMS: *m/z* (rel. int.): 272 (M^+^, 23), 271 (53), 252 (19), 243 (30), 217 (52), 230 (100), 189 (42), 179 (48); HREIMS: 272.1776 (C_18_H_24_O_2_^+^, calc. 272.1772); ^1^H- and ^13^C-NMR: see Table 1.

### 3.5. 3β-Hydroxy-5,6-dehydrosugiol (**2**)

Yellowish amorphous solid; [α]^26^_D_ +26.3° (*c* 0.23, CHCl_3_); UV (MeOH) *λ*_max_ (log *ε*): 219 (4.20), 245 (4.30), 317 (4.11) nm; IR (KBr) *ν*_max_: 3363 (OH), 1639 (C=O, conjugated), 1613, 1587, 1567, 1502, 1461, 1328, 1036 cm^−1^; EIMS: *m/z* (rel. int.): 314 (M^+^, 100), 299 (19), 281 (32), 271 (9), 258 (54), 257 (75); HREIMS: 314.1879 (C_20_H_26_O_3_^+^, calc. 314.1883); ^1^H- and ^13^C-NMR: see Table 1.

### 3.6. Chemicals

LPS (endotoxin from *Escherichia coli*, serotype 0127:B8), Carr (type IV), indomethacin, MTT (3-[4,5-dimethylthiazol-2-yl]-2,5-diphenyltetrazolium bromide) and other chemicals were purchased from Sigma Chemical Co. (St. Louis, MO, USA).

### 3.7. Cell Culture

A murine macrophage cell line RAW264.7 (BCRC No. 60001) was purchased from the Bioresources Collection and Research Center (BCRC, Hsinchu, Taiwan) of the Food Industry Research and Development Institute (Hsinchu, Taiwan). Cells were cultured in plastic dishes containing Dulbecco’s Modified Eagle Medium (DMEM, Sigma, St. Louis, MO, USA) supplemented with 10% fetal bovine serum (FBS, Sigma) in a CO_2_ incubator (5% CO_2_ in air) at 37 °C and subcultured every 3 days at a dilution of 1:5 using 0.05% trypsin-0.02% EDTA in Ca^2+^-, Mg^2+^-free phosphate-buffered saline (DPBS).

### 3.8. Cell Viability

Cells (2 × 10^5^) were cultured in 96-well plate containing DMEM supplemented with 10% FBS for 1 day to become nearly confluent. Then cells were cultured with compounds **1**–**5** in the presence of 100 ng/mL LPS (lipopolysaccharide) for 24 h. After that, the cells were washed twice with DPBS and incubated with 100 μL of 0.5 mg/mL MTT for 2 h at 37 °C testing for cell viability. The medium was then discarded and 100 μL dimethyl sulfoxide (DMSO) was added. After 30-min incubation, absorbance at 570 nm was read using a microplate reader (Molecular Devices, Sunnyvale, CA, USA).

### 3.9. Measurement of Nitric Oxide/Nitrite

NO production was indirectly assessed by measuring the nitrite levels in the cultured media and serum determined by a colorimetric method based on the Griess reaction. The cells were incubated with different concentration of samples in the presence of LPS (100 ng/mL) at 37 °C for 24 h. Then, cells were dispensed into 96-well plates, and 100 μL of each supernatant was mixed with the same volume of Griess reagent (1% sulfanilamide, 0.1% naphthylethylenediamine dihydrochloride and 5% phosphoric acid) and incubated at room temperature for 10 min, the absorbance was measured at 540 nm with a Micro-Reader (Molecular Devices).

### 3.10. Statistical Analysis

IC_50_ values were estimated using a non-linear regression algorithm (Sigma Plot 8.0; SPSS Inc. Chicago, IL, USA). Statistical evaluation was carried out by one-way analysis of variance (ANOVA followed by Scheffe’s multiple range tests).

## 4. Conclusions

Konishone (**1**) and 3β-hydroxy-5,6-dehydrosugiol (**2**) are new compounds from the wood of *C. konishii*. Compound **1** has a novel 7,20-dinorabietane skeleton. Compounds **1**, **3**, and **5** displayed a significant concentration-dependent inhibition of NO production. The compounds** 1**, **3**, and **5** may lead to the development of novel non-steroidal anti-inflammatory drugs.
